# Self-Disclosure and Spiritual Well-Being in Pastors Seeking Professional Psychological Help

**DOI:** 10.1007/s11089-017-0757-1

**Published:** 2017-04-08

**Authors:** Erik D. Salwen, Lee A. Underwood, Gabriel S. Dy-Liacco, Kathleen R. Arveson

**Affiliations:** 1 Department of Biblical Counseling, Dallas Theological Seminary, 7100 Regency Square Blvd, Houston, TX 77036 USA; 2 Regent University, Virginia Beach, VA USA

**Keywords:** Self-disclosure index, Spiritual well-being, Attitudes toward seeking professional psychological help

## Abstract

Pastoral mental health is a topic that has only rarely been researched empirically in the psychological literature, yet a pastor’s mental health can have a significant impact on churches, communities, and even nations (Royal and Thompson, *Journal of Psychology and Christianity,* *31*(3), 195–204, 2012). One of the thoughts prompting this research is that evangelical pastors might be expected to resist the findings of psychological research and lack understanding of specific mental illnesses they are potentially facing. Combined with historical and cultural dynamics that could influence resistance to professional psychological help, evangelical pastors have personal, internal factors that could also strengthen resistance, including the researched issues of self-disclosure flexibility and spiritual well-being. A correlational research design with multivariate regression was used to determine potentially significant or predictive relationships between the relevant factors. Among evangelical seminary students (*N* = 251) preparing for parish-based pastoral ministry, this research determined that no significant relationship, predictive or otherwise, existed between self-disclosure flexibility, spiritual well-being, and attitudes toward seeking professional psychological help. Implications include a shift in focus toward external factors influencing pastors’ help-seeking attitudes, such as the need for the mental health community to develop connections with evangelical pastors and the development of more support for Christian mental health professionals in the larger evangelical community.

Rarely researched empirically in the psychological literature, pastoral mental health can have a significant impact on churches, communities, and even nations (Royal and Thompson 2012). In the mid-twentieth century, certain circles of evangelical Christianity birthed a reaction to the influence of secular psychology that led to a closing of their ranks, and this reaction produced, at best, passing disdain for the field of psychology and, at worst, genuine fear among generations of evangelical pastors up to today (Carter and Narramore 1979). Instead of seeing psychological theory and praxis as resources with which to interact in order to shepherd and lead parishioners more effectively, a thorough campaign against psychology and the culture that was believed to be ‘infecting’ Christians was mounted (Adams 1970). One of the thoughts prompting this research is that, caught in this wave, evangelical pastors might be expected to resist the findings of psychological research and lack understanding of specific mental illnesses they are potentially personally facing and, furthermore, that this resistance might have a negative effect on parishioners’ help-seeking behavior.

As a result of these historical issues, pastors and their congregations have ‘doubled down’ by maintaining unrealistic expectations for pastoral leadership. “Traditional conceptions that promote unrealistic expectations of the congregation are likely in turn to foster internal unrealistic expectations kindled by a leader’s basic human need to achieve and succeed” (Ellison and Mattila 1983, p. 34). With these “internal unrealistic expectations” and a lack of awareness of potential or real impairment, the pastor will either unnecessarily resist or simply ignore the ever more competent and plentiful psychological resources available.

Combined with these historical and cultural dynamics that could influence resistance to professional psychological help, evangelical pastors have additional personal and internal issues that could also influence that resistance. One of these factors is certainly the tendency toward self-disclosure that can be rooted in one’s personality, family of origin, or other relational experiences. The very process of seeking help must involve a level of self-disclosure, and it would seem that those with a lower tendency to self-disclose would not be as willing to seek help for a problem (Hinson and Swanson 1993). Additionally, spiritual well-being, described as a subjective religious and existential sense of one’s quality of life (Moberg 1979; Moberg and Brusek 1978), would seem to relate to willingness to seek help. Spiritual well-being is specifically relevant to both the role of a pastor as well as his or her individual mental health. Being able to measure help-seeking behavior in pastors in light of their self-disclosure flexibility and their spiritual well-being would potentially shed light on factors that prevent evangelical pastors from getting the psychological help they need.

Pastors occupy a role that does not leave much room for the full range of self-disclosure as they are expected to set an example for their congregants to follow. The psychological element of self-disclosure flexibility is critical for the pastor, as an imbalance in relational connections with other people is a recipe for burnout. Chandler (2009) notes that “pastors face stress and loneliness because of a multiplicity of demands, which negatively impacts them as well as their families and constituencies” (p. 274).

In addition to the lack of self-disclosure flexibility creating relational imbalances, pastors may struggle because their spiritual well-being serves both as the foundation for their personal identity and wellness and as a vocational prerequisite. If the existential and religious elements of spiritual well-being are not sufficiently present and inextricably linked in the life of a pastor, the psychological dissonance experienced in his or her role will quickly prompt a need for help (Ellison 1983).

Struggles with other people are reflected in issues with self-disclosure flexibility, and struggles with God are reflected in issues with spiritual well-being. It would seem that any issues in self-disclosure flexibility or spiritual well-being in the life of the pastor would strongly indicate a need for – but also potentially affect the likelihood of seeking – outside help, and the relationship between these factors is the focus of the current study.

## Review of selected literature

The social sciences literature on pastoral mental health is fairly young with the first momentum being developed in the 1980s. Ellison and Matilla, in their 1983 article on the needs of evangelical Christian leaders in the United States, identify from the outset that “In recent literature, research focusing on the needs of pastors and Christian leaders has been sparse” (p. 28).

### Pastoral mental health

The early research emphasis on external professional factors and the professional tasks of pastors had a distinctly behavioral tint, but movement began toward internally generated issues, as in the case of the work by Hatcher and Underwood (1990) focusing on the connection between trait anxiety (State-Trait Anxiety Inventory), self-concept (*P* scale of the Tennessee Self-Concept Scale–TSCS), and self-criticism (Moral-Ethical subscale of the TSCS) in Southern Baptist ministers. A major driver of this movement was the realization that “healthy outcomes are more the product of effective coping than the absence of stress” (Hatcher and Underwood 1990, p. 187).

Trait anxiety had a significant negative correlation to self-concept and a significant positive correlation to self-criticism in the research conducted with 103 Southern Baptist ministers (Hatcher and Underwood 1990). With the “importance of a strong sense of self-esteem and self-efficacy to cope effectively with the demands of life” already established, the primary implication is that “a highly positive self-concept is an important resource for coping with stress” (Hatcher and Underwood 1990, p. 187). Interestingly, Hatcher and Underwood (1990, p. 192) also related their findings to “those in theological education” and made recommendations for implementing “counseling services” and “testing services.” They even recognized the reactionary perspective of “conservative, evangelical Christians” to the psychological concept of self-esteem and, therefore, recommended an intentional introduction of a “biblical basis for a positive self-concept” to address this anticipated reaction (Hatcher and Underwood 1990, p. 192).

One of the primary areas of focus in the current research is the potential correlation between intentional relationship (professional or otherwise) with others and spiritual well-being. As work on the Clergy Health Initiative continues at Duke University Divinity School, one of the qualitative studies (Miles and Proeschold-Bell 2013, p. 199) researched “the utility of peer support groups for reducing mental distress among pastors.” Although this study was very specific to United Methodist Church clergy, did not quantify any factors, and found that “participation in peer support groups had inconsistent direct and indirect relationships to mental distress (measured as mentally unhealthy days, anxiety, and depression)” (p. 199), it raised important questions about the link between personal relational competence and mental health.

Although the research of Ellison et al. (2013) was not done with pastors and did not extend out to actual measures of psychopathology, they did examine the links between spiritual struggles and psychopathology. They explain their work by stating that “divine [or troubled relationships with God] and struggles with belief are stronger predictors of psychological distress than interpersonal struggles,” and they note that their research was “interpreted in relation to previous research on . . . role theory and evolutionary threat assessment systems (ETAS) theory, which provides a plausible biological mechanism through which religious and other beliefs can directly affect mental health” (p. 215). The two accepted propositions from ETAS theory that are relevant to the 2013 study are (1) “certain classes of psychiatric symptoms . . . are the product of neural assessments of the potential threats of harm posed by animate and inanimate objects and situations” and (2) “religious and other beliefs directly affect threat assessments, and therefore psychiatric symptoms, by modulating the ETAS sensitivity to (or their threshold for) what constitutes a threat” (pp. 225–226). It is very useful to the current research that Ellison et al. (p. 225) discovered a relatively stronger positive association between “spiritual struggle (divine struggles and struggles with beliefs)” and “symptoms of depression, anxiety, phobia, and somatization” for persons describing themselves as “very religious.” The evidence that “spiritual struggles are linked with undesirable mental health outcomes among diverse samples” (Ellison et al. 2013, p. 224) provides strong support for developing a deeper understanding of how spiritual struggles in pastors might impact their help-seeking attitudes.

The current research stays solidly within the intrapersonal realm. Even in measuring attitudes toward seeking professional psychological help, no attempt is made either to predict actual psychopathologies or to correlate the intrapsychic measures with external realities. The goal of this research is to test a theoretical framework that connects spiritual well-being and a tendency to self-disclosure to a pastor’s attitude toward seeking psychological help for his or her own mental health.

### Pastoral help-seeking attitudes

In pursuing the topic of help-seeking behavior among future pastors, the final, and most important, literature category is the area of understanding more deeply pastors’ attitudes toward caring for their own mental health. This search resulted in the understanding that even though a large amount of work has been done to understand pastors’ philosophies toward giving care for others’ mental health, and a growing amount of research is being dedicated to studying how evangelical congregants maintain their own mental health, very little research has been done into pastors’ internal dynamics that reflect attitudes toward seeking help for their own mental health.

In the research that most closely approximates the current study but does not address the specific population of evangelical pastors, Royal and Thompson (2012, p. 195) use a Rasch measurement analysis to look at the reliability and validity of the ATSPPH-SF scale, specifically for 540 “Protestant Christians.” Royal and Thompson conclude that “the ATSPPH is a psychometrically sound instrument capable of producing both valid and reliable measures,” and they encourage other researchers to use the instrument “for similar purposes” (p. 203). The bottom-line result from the study is that “it appears this sample of Protestant Christians generally find it fairly difficult to seek out psychological counseling,” and the most conclusive result of the assessment was that “most people felt talking about problems with a psychologist was a poor way of getting rid of emotional problems” (p. 202).

As a practical goal of the current study is to advance the potential for positively impacting pastors’ behavior, it is encouraging that Royal and Thompson (2012, p. 203) report that “numerous researchers have found that a person’s attitude toward seeking help is a strong predictor of help-seeking behavior” (Halgin et al. 1987; McCarthy and Holliday 2004; Vogel et al. 2005). The gap in the research with pastors, especially related to their own help-seeking behavior, could have a major impact on the public and publicized missteps of pastors. This gap needs to be filled, and the current research begins to address this gap.

## Purpose

With a focus on the future health of the Church, the gap in understanding the potential impact of self-disclosure flexibility and spiritual well-being on willingness to seek help among future pastors being trained at evangelical seminaries should be explored. Accordingly, the purpose of this research is to determine the effects of self-disclosure flexibility and spiritual well-being on willingness to seek professional psychological help among evangelical seminary students preparing for pastoral leadership.

## Methods

### Sample

For the purpose of achieving a substantial sample size and getting a broad representation of evangelicalism, the decision was made to survey students at evangelical seminaries in the latter stages of their training who were planning to enter into parish-based pastoral ministry. Some of these students were likely already in a pastoral role, but that question was not asked, and, therefore, the number of participants already in that role was not determined. The seminaries included in this research were determined to be “evangelical” by their self-inclusion in the Evangelical Association of Theological Field Educators. Participants were obtained using “purposive sampling,” with a list generated by the field education offices at each of the institutions. Each list consisted of students enrolled in graduate degree programs specifically preparing them for parish-based pastoral leadership (e.g. Th. M., M. Div.), and each student had to have completed at least 30 credit hours of coursework toward the degree. No quotas were established related to any demographic data, but information was collected on each student’s gender, age, race/ethnicity, marital status, and employment status.

### Hypotheses

This research reviews the relationships between self-disclosure flexibility, spiritual well-being, and attitudes toward seeking professional psychological help. Given that these three constructs have all been validated in the literature as important to one’s psychological health (Hall 1997; Hatcher and Underwood 1990; Miles and Proeschold-Bell 2013; Richmond et al. 1985; Royal and Thompson 2012), it would seem to make sense that all three would relate positively to one another, so, out of the research questions, the following four hypotheses emerge:

1. There is a significant difference in willingness to seek professional psychological help as measured by the ATSPPH Scale by the level of self-disclosure flexibility as measured by the SDI Scale (non-directional). (RQ1)
2. There is a significant difference in willingness to seek professional psychological help as measured by the ATSPPH Scale by the level of spiritual well-being as measured by the SWB Scale (non-directional). (RQ2)
3. Self-disclosure flexibility as measured by the SWB Scale and spiritual well-being as measured by the SWB Scale, when taken together, significantly predict willingness to seek professional psychological help as measured by the ATSPPH Scale. (RQ3)
4. There is a significant relationship between the combination of self-disclosure flexibility as measured by the SDI Scale together with spiritual well-being as measured by the SWB Scale and willingness to seek professional psychological help as measured by the ATSPPH Scale. (RQ4)

### Instrumentation

Three surveys, each using a 6-point Likert scale, were used, together with pertinent demographic data that included gender, marital status, birth year, etc. These surveys were the Self-Disclosure Index (SDI), the Spiritual Well-Being (SWB) Scale, and the Attitudes Toward Seeking Professional Psychological Help (ATSPPH) Scale.

One of the more widely used assessments for measuring self-disclosure flexibility is the Self-Disclosure Index (SDI) developed by Miller et al. (1983). Interestingly, for this 10-item questionnaire, a high level of internal consistency existed when tested for “the same-sex-stranger version (Cronbach’s alpha = .93 for men, .93 for women)”; “for the same-sex-stranger, the means for men (M = 15.65, SD = 9.50) and women (M = 15.04, SD = 9.37) were not significantly different” (Miller et al. 1983, p. 1236). Hinson and Swanson (1993) introduced research with college students that investigated the relationship of help-seeking behavior to the tendency to self-disclose. They determined that the “study . . . did not establish a causal link between willingness to self-disclose to a counselor and willingness to seek help,” though they called for further research and made the point that “self-disclosure provides a relevant framework from which a person’s willingness to seek help may be explored” (Hinson and Swanson 1993, p. 465).

The Spiritual Well-Being (SWB) Scale, a commonly used assessment with subscales measuring both religious and existential well-being, has helped in understanding internal and external alignments of those planning to enter vocational ministry (Dudley and Dudley 1994). The scale was first published in 1982 and has had over 1000 requests for use in research (Paloutzian and Ellison 2009). In initial testing with 100 student volunteers at the University of Idaho, the test-retest coefficient for the 20-item scale was 0.93, and the coefficient alpha was 0.89 “suggest[ing] that SWB has high reliability and internal consistency” (Ellison 1983, p. 333). In later research, two more samples showed test-retest reliability for the 20-item SWB scale above 0.85 (Upshaw 1988; Brinkman 1989), and six more samples showed coefficient alphas above 0.84 (Bufford et al. 1991 p. 57). Also, “with regard to validity, examination of the item content suggest[ed] good face validity” (Ellison 1983, p. 333), and “SWBS and its subscales . . . are positively correlated with several standard indicators of well-being, including a positive self-concept, finding meaning and purpose in life, high assertiveness and low aggressiveness, good physical health, and good emotional adjustment” (Bufford et al. 1991, p. 57). From a validity perspective, “research has . . . shown that the SWBS is a good general indicator of well-being, and is especially sensitive to lack of well-being” (Paloutzian and Ellison 2009).

The most widely used assessment for determining help-seeking behavior is the Attitudes Toward Seeking Professional Psychological Help (ATSPPH) Scale, originally offered as a 29-item multidimensional tool by Fischer and Turner (1970). Fischer and Farina (1995) developed a shortened 10-item unidimensional assessment out of the original tool that has been shown to have good psychometric properties in evaluating Protestant Christians (Royal and Thompson 2012). After 25 years of using the 29-item assessment, Fischer and Farina (1995) acknowledged the usefulness of a shortened form that unidimensionally measured only one’s attitude toward help-seeking, as “the subscales used to measure [multiple] dimensions lacked internal consistency” (p. 368). From a validity perspective, this new 10-item assessment had internal consistency in the first of two studies of “0.84 (Cronbach’s alpha), comparable to what Fischer and Turner had obtained for their full scale (that is, 0.83 and 0.86 in two samples)” (Fischer and Farina 1995, p. 370). Additionally, in the second of two studies with the shortened assessment, “the test-retest correlation with a 1-month interval between tests was 0.80 (n = 32) . . . [whereas] the 4-week test-retest reliability reported by Fischer and Turner was 0.82” (Fischer and Farina 1995, p. 371).

Extensive follow-up reliability and validity testing was done on the 10-item ATSPPH-SF (Short Form) by Elhai et al. (2008), with “support for the ATSPPH-SF’s reliability and validity, in both samples of college students and medical patients” (p. 327). Elhai et al. (2008) firmly stated that “these factor structure findings lend further credence to using the ATSPPH-SF among college students, and establish support for its use in more generalizable samples, such as medical patients” (p. 327). Additionally, “Results corroborate criterion validity evidence for the relationship between mental healthcare use and ATSPPH-SF scores for students, with similar effect sizes (Fischer and Farina 1995), and now provide evidence for such a relationship with medical patients” (Elhai et al. 2008, p. 327). As a new finding, “Results revealed support for a two-factor model,” as “the first factor deals mainly with openness to seeking mental healthcare for one’s own emotional problems, while the second factor more generally involves perceptions about the value of treatment” (Elhai et al. 2008, p. 327). So, although the earlier research by Fischer and Farina (1995) seemed to indicate that only the single factor of the overall help-seeking attitude was valid, this more recent research opens up the possibility for more detailed understanding along a two-factor model.

### Procedures

Data collection in this research was via the Internet only, using the electronic survey website SurveyGizmo.com. All participants provided data through electronic data entry using an electronic device, such as a desktop computer, laptop computer, tablet, or smartphone.

All data was completely anonymous, with no possible identification of the participants, and no existing data or records were used in data collection. Participants were induced to participate; participants from each institution were entered into a drawing for an iPad Mini to be given to one student per institution.

Since reaching pastors in their various parishes would be highly time-intensive and financially burdensome, seminaries provided a highly concentrated group of people intending to pursue pastoral ministry. The selection of which seminaries to survey related to an opportunity to connect with the Evangelical Association of Theological Field Educators. Among this group, representatives from five seminaries were willing to participate by distributing an email invitation to their students. The email invitation provided a link to a secure website that gathered seminary students’ responses to three consecutive Likert-based surveys totaling 40 items as well as some demographic questions. In order to gather as many responses as possible, the timing of the data collection needed to be in the middle of a semester, specifically avoiding beginning-of-semester disorientation, mid-semester breaks, and end-of-semester exam preparations.

### Analyses

The statistical analyses sought to support the research hypotheses of relationships existing between the three psychological constructs being measured in this study—self-disclosure flexibility, spiritual well-being, and attitudes toward seeking professional psychological help. Since these three constructs are all validated in the literature as important to one’s psychological health (Richmond et al. 1985; Hatcher and Underwood 1990; Hall 1997; Royal and Thompson 2012; Miles and Proeschold-Bell 2013), it is hoped that each will provide a basis for valid analysis in this study.

In performing the statistical analyses, the responses from the Spiritual Well-Being Scale (SWB), the Attitudes toward Seeking Professional Psychological Help Scale (ATSPPH), and the Self-Disclosure Index (SDI) were combined into three scores, one for each of the scales/indexes. The combinations were the sum of the responses from each question. In some cases, reverse scoring was required to maintain consistency. Multiple regression was used for all four research questions, as the process of combining the survey responses into the three scores produced “continuous” predictor and response variables.

To answer RQ1 and RQ2, a multiple regression analysis was used to measure whether a significant difference exists in willingness both to seek professional psychological help by the level of self-disclosure flexibility and to seek professional psychological help by the level of spiritual well-being. Following this, to answer RQ3 and RQ4, a multiple regression analysis was performed both for the purpose of theoretical prediction as well as for the purpose of determining multivariate relationship.

Additionally, the results of administering the ATSPPH Scale were analyzed to determine an initial measurement among evangelical seminary students preparing for pastoral leadership regarding their attitudes toward seeking professional psychological help. The results were measured in total and also measured by seminary and other demographic factors (gender, age, race/ethnicity, geography, income level, etc.).

## Results

### Data screening

Data screening revealed missing data that was important to hypothesis testing. Seven respondents who did not specify which school they were attending were omitted from the data, as an important consideration for the study was the potential differences in results between the five schools at which students were surveyed. One additional respondent was excluded for not disclosing birth year. A total of 251 observations across the five schools remained.

In assessing self-disclosure flexibility using the SDI, spiritual well-being using the SWB, and help-seeking attitudes using the ATSPPH, multiple questions were asked for each of the three assessments. Scores were calculated for the SDI, SWB, and ATSPPH by adding the scores of the questions within each assessment. A 6-point Likert Scale from 0 to 5 was used for all questions across all three assessments. The SDI and ATSPPH scores, with 10 questions each, had a range from 0 to 50, and SWB scores, with 20 questions, had a range from 0 to 100. Some questions had their scores reversed in order to ensure that each question’s score was measuring the outcome of interest properly (questions 1, 2, 5, 6, 9, 12, 13, 16, and 18 for the SWB; 2, 4, 8, 9, and 10 for the ATSPPH).

### Descriptive analysis

**Fig. 1:**
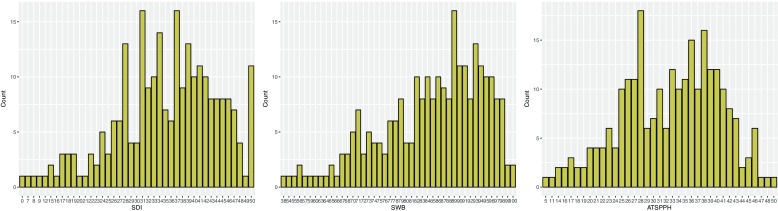
Range of scores for the SDI, SWB, and ATSPPH scales

For the SDI and ATSPPH, the possible scores ranged from 0 to 50, and, for the SWB, the possible scores ranged from 0 to 100. In the series of three bar graphs in Fig. 1, the x-axis represents the range of scores for each of the three assessments by participants in the study, and the y-axis represents the number of participants. These bar graphs are included to show the distribution of scores by participants for each of the three assessments.

**Fig. 2:**
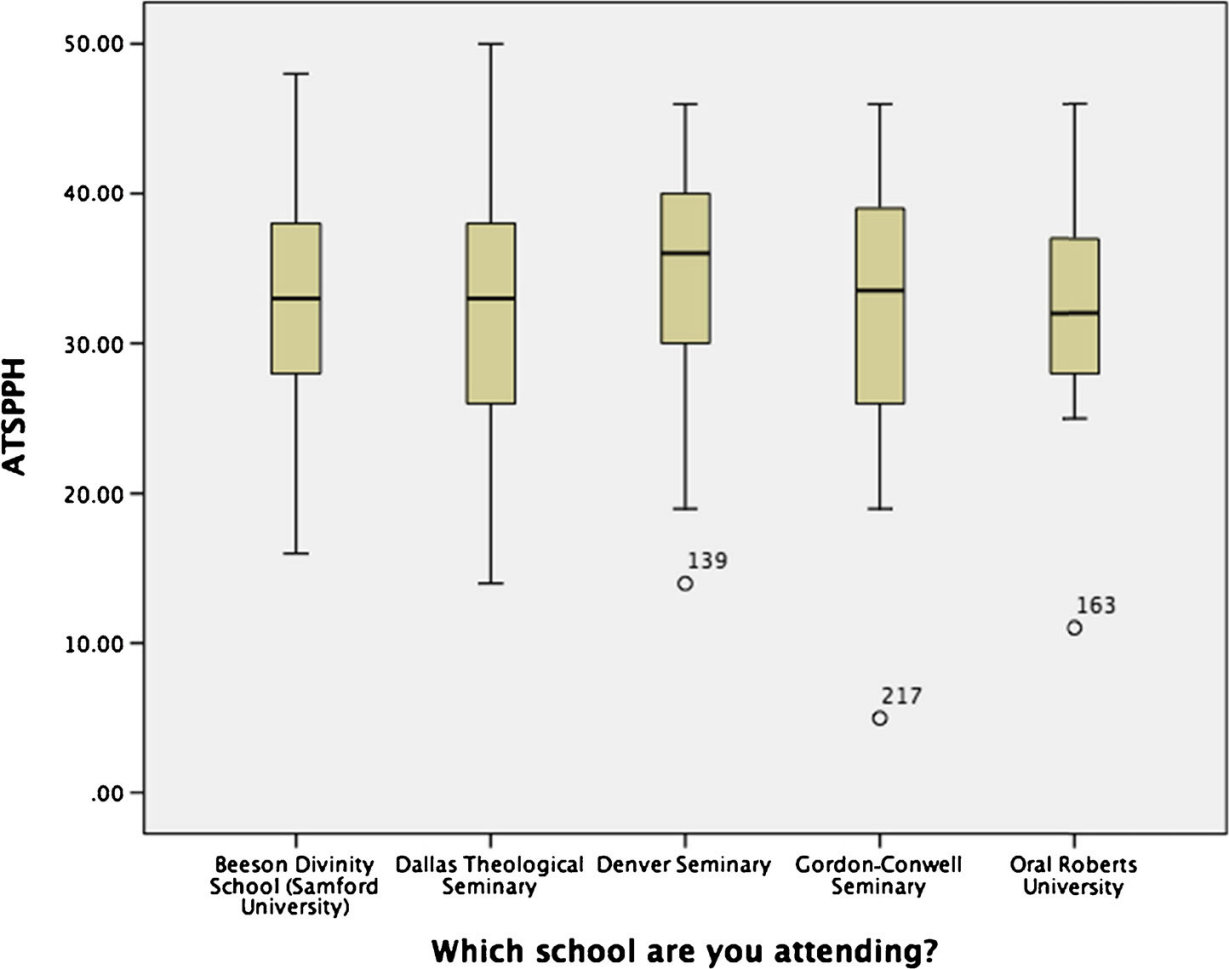
Box plot of ATSPPH scores by school attended

Also of particular interest from a descriptive perspective was the distribution of ATSPPH scores by key demographics, including school attending, gender, birth year, and race/ethnicity. In Fig. 2, the ATSPPH scores (as labeled on the y-axis) are broken out by each of the five schools (as labeled on the x-axis). The scores per school are represented on the graph with the boxes indicating the interquartile deviation and the marker through the middle of the box indicating the median score. The dotted lines or “whiskers” protruding out of the top and bottom of the boxes extend to the minimum and maximum scores on the ATSPPH by participants from that school. Additionally, Denver Seminary, Gordon-Conwell Seminary, and Oral Roberts University each have one outlier represented on the graph by the small dots on the low end of the ATSPPH scores.

### Inventory scores

A multivariate multiple regression model was fitted to the ATSPPH scores to determine how well self-disclosure flexibility and spiritual well-being predicted those scores. Due to the large number of potential predictors, backward selection using the Akaike information criterion was used to help identify important predictors and relevant interactions. Once important predictors were identified, model diagnostics were performed and the model was modified to ensure a statistically valid fit. Residual plots indicated no significant departures from the model assumptions. A Shapiro-Wilk test did not show any strong evidence against the normality assumption of the residuals (*p* = 0.17).

Predictors used in the final model included both the SWB scores and the SDI scores. Demographic variables also used as predictors in the final model included school, gender, birth year, Latino/Hispanic American, Non-Hispanic White or Euro-American, and Other/Multi-Racial. Several race variables (including Black/Afro Caribbean/African American) were not significant, which may be due to small sample sizes if such effects exist.

It is also interesting to note other variables that were not included in the final model. In addition to several race variables, marital status, employment status, and total household income (when modeled as a continuous variable) were not included through the model selection process. This, however, is not too surprising when viewing the marginal relationship of each of these variables with the ATSPPH scores.

In Table 1, the important independent variables or relevant predictors are listed with their regression coefficient estimate indicating their relative strength of prediction of ATSPPH scores. Also listed are the standard error (as a measure of the precision with which the coefficient is measured) and the t-value. Beeson Divinity School is not listed, as it is the base school category for analysis. Female gender is not listed as it is the base gender category for analysis.

**Table 1:** Regression coefficients for relevant predictors of ATSPPH scores

Coefficients	Estimate	Std. Error	t Value
(Intercept)	−1.222e + 02	1.251e + 02	−0.977
SWB	−4.421e − 03	4.916e − 02	−0.090
SDI	9.152e − 02	5.400e − 02	1.695
Dallas Theological Seminary	−5.706e − 01	1.473e + 00	−0.387
Denver Seminary	1.066e − 00	1.629e + 00	0.655
Gordon-Conwell Seminary	2.005e − 01	1.541e + 00	0.130
Oral Roberts University	2.190e − 02	2.209e + 00	0.010
Male	−3.381e + 00	1.364e + 00	−2.478
Birth Year	7.702e − 02	6.276e − 02	1.227
Non-Hispanic White or Euro-American	2.627e + 00	1.295e + 00	2.028
Latino or Hispanic American	8.577e + 00	2.872e + 00	2.987
Other/Multi-Racial	−5.138e + 00	3.248e + 00	−1.582

The linear combination of the above predictors was not significantly related to ATSPPH scores, *F*(11, 239) = 2.33, *p* < 0.01. The sample multiple correlation coefficient was 0.31, indicating that approximately 10% of the variance in ATSPPH scores in the sample can be accounted for by the linear combination of the above predictors. In Table 2, the *p*-values are calculated for the same important independent variables or relevant predictors in order to provide the most meaningful statistic regarding the probability that any of the variables are having an effect.

**Table 2:** Probability of marginal significance for predictors of ATSPPH scores

*p*-values	Pr (>t)
(Intercept)	0.32957
SWB	0.92843
SDI	0.09141
Dallas Theological Seminary	0.69889
Denver Seminary	0.51322
Gordon-Conwell Seminary	0.89657
Oral Roberts University	0.99210
Male	0.01390*
Birth Year	0.22094
Non-Hispanic White or Euro-American	0.04368*
Latino or Hispanic American	0.00311**
Other/Multi-Racial	0.11507

Significance codes: ***p < 0.001, **p < 0.01, *p < 0.05, .*p* < 0.1

Neither the SWB (*p* = 0.93) nor the SDI scores (*p* = 0.091) were significant. Additionally, none of the schools were significantly different from Beeson Divinity (the base category for analysis), as all *p*-values for each of the other four schools were greater than 0.5. Two race/ethnicity variables were significant, Latino/Hispanic American (*p* = 0.003) and Non-Hispanic White or Euro-American (*p* = 0.04), both of which had higher ATSPPH scores on average (8.6 and 2.6, respectively). The Other/Multi-Racial category was not statistically significant (*p* = 0.12). Men scored an average of 3.4 points lower than women (*p* = 0.01), though birth year was not significant (*p* = 0.22).

A model reduction F test was also performed to assess only the joint statistical importance of SDI and SWB scores in predicting ATSPPH scores. The combination of SDI and SWB scores was not significantly related to ATSPPH scores, *F*(2, 243) = 1.62, *p* = 0.20.

## Discussion

This study sought to understand the dynamics and relationship between three internal factors of evangelical seminary students preparing for parish-based pastoral ministry: self-disclosure flexibility, spiritual well-being, and willingness to seek professional psychological help for one’s own mental health. The clear finding of this research was that no significant relationship exists between the three factors. The instruments measuring self-disclosure flexibility, spiritual well-being, and willingness to seek professional psychological help for one’s own mental health are intended to measure across the poles, and the lack of significant relationship was regardless of whether the results on any of the three instruments were high or low. This finding suggests that other elements might play a larger role in a pastor’s willingness to seek help for his or her own mental health. Focusing on the future health of the Church, the gap in understanding the potential impact of self-disclosure flexibility and spiritual well-being on willingness to seek help among future pastors being trained at evangelical seminaries needs to be explored and filled. Accordingly, the purpose of this research was to determine the effect of self-disclosure flexibility and spiritual well-being on willingness to seek professional psychological help among evangelical seminary students preparing for pastoral leadership.

### Implications

Although the lack of relationship between self-disclosure, spiritual well-being, and attitudes toward seeking professional psychological help was not anticipated based upon initial hypotheses, implications do emerge from this study regarding the maximization of pastoral mental health. Given the moral gravity, visibility, and influence of the pastoral role, maintaining mental health in such a role seems to be a must. Other studies have looked, on the one hand, at many different ways pastors are at risk (Hall 1997) and, on the other hand, at many different ways pastors can engage in appropriate processes of maintaining wellness (Proeschold-Bell et al. 2011). Whether avoiding the risks or enhancing wellness, the constituencies invested or involved in pastoral mental health should consider what might be learned from this study. Implications from this research need to be considered regarding pastors of evangelical churches, clinical mental health professionals, and the evangelical community as a whole.

#### Pastors of evangelical churches

As spiritual well-being and self-disclosure flexibility are neither related to nor predictive of attitudes toward seeking professional psychological help among evangelical seminary students preparing for parish-based pastoral ministry, what does this mean? This finding certainly does not mean that either spiritual well-being or self-disclosure flexibility is unimportant in the life of the pastor. However, it may mean that attitudes toward seeking professional psychological help have more complex origins in social and cultural factors relating to the perception of professional psychology, such as denominational alignments, individual pastoral and mentoring relationships, and exposure to a nouthetic counseling philosophy.

Still, this study clearly found that, in moments of spiritual crisis, weakness, stress, or struggle, these factors do not necessarily have an impact on the willingness of the pastor to seek professional psychological help. This is not completely surprising, as spiritual struggles do not always indicate mental health problems. Spiritual struggles, in a more religious sense, can stem from currently difficult external circumstances that lead the pastor to move into a mode where he or she is functionally operating as though God is not present or able to help—essentially taking control of whatever situation presents itself. As these religious struggles would typically be more externally or circumstantially generated, a pastor could decide to seek professional help for an adjustment issue, but this study does not indicate that this is a natural conclusion for the pastor to make. Spiritual struggles, in a more existential sense, can stem from deeper internal doubts or questions that may actually lead the pastor to question whether he or she even believes in God, and, therefore, whether he or she ought to remain in pastoral ministry. As these existential struggles would typically be more internally and pervasively generated, a pastor may be helped much more profoundly by a mental health professional, but, again, this study does not indicate that the pastor will reach this conclusion. Why would the pastor, who has been convinced in his or her education and experience that he or she already occupies the most spiritually informed vocational role, bring his or her spiritual struggles to someone in a different role?

On the other side of the equation, a pastor may enjoy a high level of religious and existential spiritual well-being but may come up against internal or external dynamics that challenge that well-being. In exercising that spiritual well-being, the pastor may, instead of seeking professional help, simply spend more time in the spiritual disciplines—prayer, reading and meditating on the Scriptures, fasting, etc. The implication from the research is that neither low nor high levels of spiritual well-being will make the evangelical pastor more or less willing to seek professional psychological help.

The evangelical community does have many leaders who have personally benefitted from professional mental health services and others who have actually co-located professional counseling centers at their churches. One implication of this study is that these pastors have an opportunity to lead the way for their colleagues who have not personally experienced this care or provided such a resource in their churches. Another implication of this study is that evangelical seminary educators should consider placing greater emphasis on Christian counseling in the pastor’s training—both for his or her congregation and for himself or herself. Some secondary or extraneous variables that were not measured in this study but that may have an impact include personality type and overall attitude toward the mental health community as a resource for the church. Pastors need to understand that professional mental health resources can align with and complement what they understand to be the Church’s primary biblical tasks of evangelism and discipleship.

It is also interesting that a higher or lower tendency toward self-disclosure also does not correlate with or predict pastors’ greater or lesser willingness to seek professional psychological help. This finding is potentially at odds with the conclusion of Hinson and Swanson (1993), who wrote that “one’s initial reservations about self-disclosing to a counselor may be disregarded in the face of one’s urgent need to obtain relief from suffering” (p. 468). The implication—and primary finding—of the current study is that even if a pastor is suffering from a low level of spiritual well-being and has a low tendency toward self-disclosure, this is still not predictive of a greater or lesser willingness to seek professional help. Perhaps the pastor is willing to self-disclose upon realizing an urgent need for relief, but he or she does not make a distinction between a professional and a non-professional counseling resource. From this discussion, it seems clear that implications also exist for mental health professionals in how they connect with evangelical pastors regarding the pastors’ own mental health.

#### Clinical mental health professionals

Clinical mental health professionals have an opportunity to play a critical role in helping pastors to achieve the greatest possible level of wellness as these helping professionals promote alignment of the pastor’s personal values and morals with his or her own behavior. Given the development and broader acceptance of a model of Christian professional care in today’s culture, it is not out of the question for the Christian counseling community to take some very specific steps to connect to evangelical pastors. One specific example of how this could happen is through the “Pastoral/Theological” representative on the Board of Directors of the Christian Association for Psychological Studies (CAPS). As a group of “Christian mental health professionals,” CAPS has a stated goal of “strongly encourag[ing] local area activities which provide networking and fellowship opportunities” (http://caps.net/distinctives). At the local chapter level, CAPS could sponsor workshops for counselor educators to reach out to the pastors in the community and train them in bringing effective care into their ministries. Training could relate to content areas such as grief, trauma, and marriage and family issues. Training could also involve focus on how a pastor might launch and support a counseling ministry in his or her church.

One implication of this study is that, within evangelicalism, it will take proactive work on the part of the clinical mental health community to create a greater connection with the churches. It is already known that a number of evangelical seminaries are decidedly against a professional model of providing counseling, though none of those are represented in the current study. These schools are currently producing pastors who have been taught that “biblical sufficiency” means the pastor not only has nothing to learn from psychological theories, methods, and techniques but also should resist and reject them. The finding that internal factors such as self-disclosure flexibility and spiritual well-being do not predict attitudes toward seeking professional psychological help leaves open the possibility that other influences are significant factors. That said, many pastors convinced of “biblical sufficiency” in their seminary training are going out into ministry and are finding that they must expand their knowledge of how to care for both their congregations and themselves.

#### Greater evangelical community

Currently, the greater evangelical community is a mixed bag in terms of its approach toward counseling. Some evangelical churches have a strict commitment to a nouthetic approach and would never dream of engaging with a mental health care professional. Some are more open to professional counseling, but they choose to quietly refer people, when the pastor feels the issue is beyond his or her capability. Others more openly engage with the professional counseling community or even have professional counselors on their paid staff.

In almost all cases, though, the decision of what approach to use is driven by pastoral leadership. Mental health care professionals will be found in many of these congregations, and these people, as part of the greater evangelical community, need to take responsibility for engaging with their pastoral leadership in promoting a model of care that honors evangelical doctrine while remaining open to what can be learned from psychological science. If anything, a primary implication of this study is that the organic factors one might hope would lead a pastor to seek professional help are not doing so, and ways of proactively connecting pastors with the mental health community from all directions should be explored.

### Future research

A starting point for future research should involve exploring secondary or extraneous variables that were not measured in this study but that may have an impact on pastors’ attitudes toward seeking professional psychological help. These variables could include pastors’ personality types, perspectives on “Christian” professional mental health services, and pastors’ overall view of the value of professional mental health services for their congregants.

Future research could broaden the scope of the research program employed in this study to seminaries outside of evangelicalism to potentially discover differences between types of Christian churches. Roman Catholics or more liberal mainline Protestant churches could present a different profile regarding the factors studied. The training process, hierarchy, and implementation of confession as a sacramental and ritualistic practice could all be factors influencing responses from Roman Catholic priests to differ from the findings in this study. The more egalitarian approach to women in pastoral leadership as well as more liberal perspectives on social and moral issues, particularly in the areas of marriage and sexuality, could cause the more liberal churches also to respond differently. These differences, as they relate to leaders’ help-seeking attitudes, could be compared and contrasted in a future study.

Another area of future research within the seminaries would be to implement a research program across seminaries with academic programs that include professional counseling and seminaries with academic programs only in pastoral counseling. The research program could be implemented with both students preparing for pastoral ministry as well as those students preparing for counseling ministry. Such research could provide insight into how some future pastors (and future pastoral counselors) may be receiving indoctrination for or against the use of professional mental health services. Perhaps a quantitative quasi-experimental research design would be helpful by relating the experience of the professional counseling students to that of the pastoral counseling students.

Since the focus of this study was on students intending to enter pastoral ministry, future research could also seek to extend the same research program to those who are already operating in the field as pastors of churches. Perhaps a measure of experience in the field by pastors post-graduation could bring a different perspective on personal use of counseling resources. Still, this type of future research may be more difficult to implement, as many denominations are looser in their organization and broadly dispersed, so gaining a representative sample of the different flavors of Christian churches could be difficult. Yet, the benefit of such a study could be to validate the findings of this research and even possibly determine differences between specific denominations.

Not knowing exactly how to recruit for such a study, future phenomenological research with pastors who have suffered public disgrace as a result of mental health-related issues could provide critical insight into any future quantitative efforts at evaluating factors precipitating such a fall and could even determine the more common clinical diagnoses that might provide predictive insight. A qualitative study examining pastoral moral failures might be used to understand how the mental health community could develop processes and programs to identify and address narcissism and sexual addiction.

If it is possible to survey a broad representation of pastors already in church-based ministry, future research should also be conducted by building a profile regarding the pastors’ use of professional counseling resources. Profiling questions would include the use of a professional counseling referral list, sponsoring of a professional counseling resource located near (but not on) the church site, co-location of a professional counseling resource on the church site (legally separate), and integration of a professional counseling ministry inside the church (legally joined). This research could also extend beyond the pastors to those who attend churches implementing different models of resourcing professional mental health care.

Although this research did represent a high level of geographic variety, a greater concentration of participants was located in the Southeastern portion of the United States. Gordon-Conwell Seminary is located in Massachusetts and Denver Seminary is located in Colorado, but the West Coast, Southwest, and Midwest were largely unrepresented. Future research should fill the geographic gaps to allow for comparison of the geographic regions and determine any potential differences. Additionally, extension of this research internationally could reveal differences across countries.

Given the finding of a significant difference in help-seeking attitudes between male and female pastors, it would make sense to prioritize future research into potential factors that make female pastors more likely to seek professional psychological help. Determining these factors could provide great insight into ways that male pastors might be encouraged to join their female counterparts in seeking help. Future research could also be conducted regarding the issue of gender to show how female pastors are potentially better able to lead in certain areas of ministry.

Because significant differences were also found between racial and ethnic groups, future research should be conducted to develop a clearer picture of the challenges facing different groups. With the much higher level of self-segregation in churches compared to other common societal structures, differences across races and ethnicities in the pastorate could have a disproportionate effect on the different churches. Within evangelicalism, what are the factors that make African American pastors less likely to seek professional psychological help than Euro American or Hispanic American pastors? Are the issues specific to expectations of the pastor in those racial or ethnic groups or are they generalizable out to the group as a whole? It also is curious that “Latino/Hispanic Americans” in general are less likely to seek professional psychological help (Fraga et al. 2004, p. 54), whereas evangelical “Latino/Hispanic Americans” in this study belonged to the racial or ethnic group most likely to seek professional psychological help. Future research should be conducted to determine the factors creating this difference.

## Conclusion

The Church is a spiritual and a human institution. Spiritually, it is understood that Jesus Christ “is the head of the body, the church” (Col. 1:18, ESV). Humanly, the pastor is supposed to “shepherd the flock of God that is among you, exercising oversight, not under compulsion, but willingly, as God would have you; not for shameful gain, but eagerly; not domineering over those in your charge, but being examples to the flock” (1 Peter 5:2–3). This picture painted of a pastor is one that demonstrates humility, maturity, and a measure of mental health that requires self-awareness and a deep spiritual connection to God. When pastors ignore the critical human and spiritual resources God has placed around them, such as Christian counselors, they do so at their peril.

Moving forward from this study, it is helpful to understand the lack of relationship between self-disclosure flexibility, spiritual well-being, and attitudes toward seeking professional psychological help, but this result only highlights the need for more understanding of how to maintain pastoral mental health. This result only opens the door to many other possible ways to draw the connection between Christian counselors with amazing abilities to provide care and pastors who often need the care these counselors can provide.
